# Robotic surgery: India is not ready yet

**DOI:** 10.4103/0970-1591.33443

**Published:** 2007

**Authors:** Girish G. Nelivigi

**Affiliations:** Dept. of Urology, KEM Hospital, Parel, Mumbai - 400 012, India

**Keywords:** Robotic surgery, radical prostatectomy, telepresence

## Abstract

Robotic surgery is one of the most significant advances in urology in recent years. It promises to make urological surgeries safer with far superior results as compared to laparoscopic or open surgeries. It holds great promise for the surgeons and patients alike. However like any other technological advance, it too comes with a heavy price tag. Aggressive marketing by the manufacturers and urologists may lead to unethical practices. This article analyses the applicability of robotics to urology and India in particular taking into consideration the financial aspects involved. At present, the scope for robotics in India is limited because of cost considerations. The future of robotic surgery in India also will depend on the same factor.

We live in exciting times. We have witnessed in our lifetime technological advances that were the stuff of science fiction a few decades ago. Nanotechnology, cloning, genomic mapping, minimally invasive surgeries, genetically altered food…. the list is endless. Robotics in surgery is one such emergent technology which is creating waves in North America and Europe. India too is not left behind with at least three centers in India acquiring robots in their surgical departments. This is an appropriate time to discuss the evolution of robotics, their transition in medical field, their utility value and their relevance or otherwise to the medical scenario in India.

## EVOLUTION OF ROBOTS

The genesis of robots goes back to the 1980s when a group of researchers at the NASA collaborated with engineers of the Stanford Research Institute. The first effort of this combined venture was the development of a ‘Telepresence’ surgical system to improve dexterity in microscopic hand surgery.[1]

The US Department of Defense soon got interested in this technology. The Military wanted a remote-operated surgical system wherein the wounded in the battle could be operated upon by surgeons away from the frontline. This was called as the SRI Telepresence Surgery System.

The SRI system was intended to be a battlefield surgical system for combat casualty care in which a mobile robotic system remote-controlled by a surgeon could do temporizing, lifesaving vascular surgeries. Although this concept could not be further developed in the military, it led to the eventual development and marketing of the da Vinci Surgical System.

A prototype robot was developed by Nanyang Technological University of Singapore in the 90s called Urobot meant for limited uses in laser prostatectomies.[2] Johns Hopkins University developed PAKY, a robotically controlled device for percutaneous access to the kidney.[3] However ROBODOC hip replacement milling device was the first robotic surgical device to be marketed in 1992. The first robotic resection of tissue in humans was done in March 1991 by PROBOT in London when it was used for TURP.[4]

The da Vinci Surgical System was released in April 1997 and received FDA approval in 2000 for laparoscopic surgeries.

Today the da Vinci surgical system is being used in urology, cardiothoracic surgery orthopedics and general surgery.[5] It has also been used in gynecology for tubal ligation reversal.

With this background, let us discuss the role of robotic assisted surgeries in surgical practice.

## ADVANTAGES OF ROBOTS

Let us discuss in detail about the purported advantages of robots and see whether the arguments made by the manufacturers and proponents of robotic surgery are valid.

1) The most common argument in favor of robots is that they make the surgery minimally invasive. But minimally invasive surgery is not an inherent property of robotics. Minimally invasive surgery can be done and is being done all over the world by surgeons without recourse to expensive robots. The truth is robotics is only one of the ways of doing minimally invasive surgery.

2) Robotics makes surgeries extremely precise by eliminating hand tremors and movement scaling resulting is fewer errors.

True. Robots eliminate tremors and help in movement scaling. However, these impressive facts hide a truth. Modern surgeries fail not because of tremors or awkward surgeons’ hand movements but because surgical treatment has its own limitations. The results of surgery depend on patient factors, tissue factors, infection and the incorrect application of surgery to an individual patient.

Modern optics, suture materials and instruments have resulted in unparalleled technical finesse in surgeries. CABG failure due to surgeon's tremors or awkward hand movements is very rare. It is more likely to fail because the original disease of the patient is left untreated. A renal transplant rarely fails if the vascular anastomosis is done well. It commonly fails because of the human immune system. Even a perfectly computer matched and milled hip replacement fails because of mechanical wear and tear of the device after a few years.

Is extreme precision needed in a majority of the surgeries? Let us take the example of radical prostatectomy. Preserving neurovascular bundle needs precise dissection. Laparoscopic surgery achieves a neurovascular bundle saving rate of close to 90%.[6] The most impressive results in radical prostatectomy in terms of potency and sphincter preservation are from Detroit. However, these superb results of VIP Technique are mainly due to a major change in technique by preserving the Veil of Aphrodite rather than due to robotic surgery per se.

3) Robots reduce the learning curve for laparoscopic surgery.

This claim is very appealing to all surgeons who are beginning their surgery careers with minimum or no laparoscopic surgery training. The majority of surgeons who are doing robotic surgery today are laparoscopy-trained and hence it has not been proved whether it is true or not. However, according to Mani Menon *et al.*, it is unclear whether Robotic Radical Prostatectomy reduces the learning curve for laparoscopic surgery.[7]

4) Robotic surgeries are ergonomically superior and cause less fatigue for surgeons in prolonged surgeries.

Yes. It is true. However, the majority of surgeons are used to operating with minimal fatigue for three to four hours. Fatigue is a factor only in surgeries longer than this. There are very few urological laparoscopic surgeries which get prolonged to such extended time. Moreover, is any urologist prepared to spend 1.4 million dollars for a comfort which can be experienced in less than 10% of his surgeries?

## DISADVANTAGES OF ROBOTS

Cost: da Vinci system costs $ 1.4 million (Rs. 7 crores) and annual maintenance costs of $ 100,000 with a lifespan of five years.[8] In the same study it was estimated that robotic surgery increases the cost of ASD closure by $ 3773 and by $ 3444 for mitral valve replacement.

A study by Louis Kavoussi is even more illustrative. He compared the cost difference between robotic pyeloplasty and laparoscopic pyeloplasty. The cost of robotic pyeloplasty reaches $9500 if < 50 cases are done per year and it needs a minimum of 500 cases per year to be comparable to the cost of laparoscopic pyeloplasties. The reduced surgery time in robotic surgery is more than offset by increased ‘setup’ and ‘takedown’ times for robots.[9]

Lotan *et al*. from the University of Texas too compared the cost difference in conventional retropubic radical prostatectomy, laparoscopic prostatectomy and robotic prostatectomy and found that the conventional retropubic radical prostatectomy was the most cost-effective with cost advantages of 487 dollars and 1726 dollars over laparoscopic prostatectomy and robotic prostatectomy respectively. This cost difference could not be compensated even by shorter operative time and shorter length of stay per patient.[10] Hence it is clear that robotic surgery is not cost-effective.

### Are robots more effective than laparoscopic surgery?

Robotic surgery is essentially another way of doing laparoscopic surgery albeit with better technical inputs and technology. This is borne out by the numerous publications that have examined this issue across various specialties.

Guy Vallencian, who has one of the biggest experiences in laparoscopic radical prostatectomy in the world, opines that an experienced laparoscopic surgeon can get better results with endo-suturing than a robotic surgeon with lesser surgical experience. He also opines that the robotic 3D Vision is not an added advantage but an indispensable tool of the telemanipulator because it is the only way for the operator to know the exact position of the instruments in the operation field.[6] In another study it was found that there is no reduced operative time, improved postoperative course and functional results.[11]

In fact in a series of robot-assisted splenectomies, it was found that robot-assisted splenectomy patients fared worse than conventional laparoscopic splenectomies.[12] Similarly, robotic adrenalectomy did not improve perioperative quality of life when compared to laparoscopic adrenalectomy.[13] Robotics in thoracic surgery have not improved the steep learning curve of laparoscopic surgery.[14]

There are concerns from the anesthetic point of view also. Mariano *et al*. reported that robotic surgery severely restricted patient access due to the bulky and space-occupying device.[15]

These apart, there are major medico-legal problems which have to be addressed. A robot after all is a computer and has the same problems that a computer faces. In the era of deadly computer viruses it is possible for any prankster to rewrite the program so that the robot refuses to obey the surgeon's commands or worse still, carry out an entirely different and unintended task. Who will be responsible for these mishaps—the surgeon, the hospital or the robot manufacturer? In fact this sort of problem already seems to be playing out in Germany where a group of patients have sued Integrated Surgical Systems Inc. the manufacturers of Robodoc for being ‘defective and dangerous'.[16]

A fact which is ignored by most of the people is that a robotic surgery needs two trained surgeons. One to insert the ports and the second surgeon to operate at the console. In case the console is situated far from the OT then the scrub surgeon should be capable of taking over the surgery and completing it. So a thorough laparoscopic training for at least one of the surgeons is a must. This negates the strong argument of the proponents of robotics that laparoscopic training is not essential to do robotic laparoscopic surgeries.[17]

## ROBOTIC SURGERY AND THE MARKET FORCES

It is clear from the preceding discussion that robotic surgery has equivalent or better results in some surgeries but for a majority of surgeries across various specialties, it offers no real patient benefits in terms of surgical outcomes. In addition it greatly increases the cost of surgery. Despite this it is so aggressively marketed as The Next Big Thing. Why is it so?

Like most of today's world, the healthcare sector too is driven by the market forces. The market forces in the healthcare industry are the Pharmaceutical companies, Instrument and Equipment manufacturers. Robotic surgery is only one of the new hyped technologies which have been aggressively pushed by the industries. The list of such new technologies which have failed after the initial hype is long. In their book, Hope or Hype: The Obsession with Medical Advances and the High Cost of False Promises, Drs. Richard A Deyo and Donald Patrick examine various such case studies where the patients have been taken for a ride. Among the many examples they quote:

Researchers in 2002 found that new and expensive anti-hypertensive drugs were less efficacious than the old and cheap diuretics. However, thanks to the aggressive marketing of the newer drugs hardly anyone uses diuretics anymore.

In 2001, the New England Journal of Medicine reported that a new therapy for breast cancer was no better than the standard cure even though it was twice as costly and more toxic. By that time however 42,000 women already had spent 3.4 billion dollars on the new treatment.

They further write in their book that bad money trumps good science. A majority of the ‘breakthroughs’ are only ‘Me Too’ technologies without any real benefit. However, these things go unnoticed for a long time because the public make certain assumptions about the medical industry. They are:

1. Doctors adopt medical sciences only on the basis of good science.
2. Newer is always better.
3. More medical tests are always better.
4. Action is always better than inaction.

We know that these assumptions are not always true. Most of us are cleverly misled by the drug companies and equipment manufacturers as we are not trained researchers. In a two-pronged attack, the pharma companies target the doctors who prescribe the drugs and by direct consumer marketing, brainwash patients into demanding prescription drugs from their doctors.

Even the American FDA which is considered by many doctors to protect patient interests has strong prejudices in favor of the industry. In the recent Vioxx fiasco, the FDA committee which voted to approve Merck to market the drug had 10 of the 32 committee members on the payroll of the drug industry.[18]

It is evident that robot-assisted surgeries do not offer any significant advantage as compared to laparoscopic surgery. Even in the affluent West, cost constraints play an important role in deciding on the acquisition of any new system. Now let us examine the role that robotic surgery can play in India. For any technology to be grafted in India, it has to take into account the unique socioeconomic conditions in India. Table 1 compares the health statistics of various countries and illustrates that robotic surgery is still a distant dream in India.

**Table 1:** Health statistics of countries-a comparison[19–22]

	USA	UK	France	Germany	India
Per capita income (US Dollars)	33070	24486	22751	23534	441
Per capita public health spending (USD)	2051	1429	1785	2063	4
Per capita health spending (USD)	4271	1675	2288	2697	< 4
Health expenditure (% GDP) (Year 2001)	13.9	7.5	9.4	10.8	5.2
Beds per 1000 population	2.8	3.7	3.8	6.6	0.8
Severely underweight under 5 pop. %	< 1	< 1	< 1	<1	18

India also has sorry disease and mortality statistics. Tuberculosis kills 500,000 Indians each year. Malaria affects 2.6 million people each year and killed at least 20,000 people in 1999. One hundred thousand Indian women die of pregnancy-related causes each year.[23]

The list of statistics is endless and mind-numbing. The question is: Can we afford it? To answer this question we must look at the way in which health services are structured in our country. Eigty per cent of the health services are provided by the private sector even though 26% of the population of our country lives below the poverty line. The government cannot afford any new and costly technologies in public hospitals. The private health sector can afford new and expensive technologies but as happens with any market-driven force will try to force the new but at times unnecessary technology upon an uninformed patient. If doctors strictly stick to the indications for using robotic surgery in select patients, it is clear that the investment cannot be recovered. Hence robotic surgery will be marketed as a panacea for all diseases requiring surgery. This in turn will make medical treatment so expensive and drive up the premiums for health insurance making health insurance unaffordable to a huge majority of our population. Also such hype will change indications for surgeries and ‘popularize’ some surgeries. This scenario is already happening in the US where for the first time in its history, live donor nephrectomies have exceeded the number of cadaveric kidney donations![24] This came about because of very aggressive marketing of minimally invasive donor nephrectomies by the doctors and medical industry alike. If this can happen in the US where literacy and consumer awareness is so high and the laws on ethics are so stringent, one shudders to think what can happen in India.

The phrase ‘Robot Surgery’ is so catchy that it is very easy to sell this concept to the unsuspecting public. After a demonstration of robotic surgery at Hyderabad, the Rajya Sabha MP K. Rama Mohana Rao who was a guest at the event demanded in the Parliament the installation of the da Vinci system in all the major government hospitals in India, keeping in mind its usefulness.[25] If someone as aware as a parliamentarian can be so much impressed, what will be the plight of so many Indians who are so illiterate that they ask no questions before undergoing any surgical procedure and for whom informed consent is an abstract concept?

Recent history is replete with many technologies which were hyped up but later on failed to live up to their promise as the progress in their research was not up to the mark. Gene therapy, immunotherapy, Lasers, TUNA, various surgeries for SUI, etc. have not lived up to their hype. After initial enthusiasm, questions are being asked about drug eluting stents in cardiology. So is robot-assisted surgery a new surgical miracle out to change the way surgery is practiced on the earth or is it one of the countless ‘Me Too’ technologies that came, were seen but never could conquer? Only time will answer this question But to quote Alexander Pope, “Be not the first by whom the new are tried, nor yet the last to lay the old aside”. Robotics is still an evolving technology with no great benefits to justify its cost. Let us put wisdom before knowledge and wait for more time to see its real benefits once the initial euphoria over this technology fades. Let us remember, a fool learns from his own mistake, whereas a wise man learns from others’ mistake!
